# AI vs. AI: Can AI Detect AI-Generated Images?

**DOI:** 10.3390/jimaging9100199

**Published:** 2023-09-28

**Authors:** Samah S. Baraheem, Tam V. Nguyen

**Affiliations:** 1 Department of Computer Science, Umm Al-Qura University, Prince Sultan Bin Abdulaziz Road, Mecca 21421, Makkah, Saudi Arabia; 2 Department of Computer Science, University of Dayton, Dayton, OH 45469, USA; tamnguyen@udayton.edu

**Keywords:** GAN-generated images detection, GAN image localization, detection of computer-generated images, fake AI-generated images recognition, fake and real detection, convolutional neural networks

## Abstract

The proliferation of Artificial Intelligence (AI) models such as Generative Adversarial Networks (GANs) has shown impressive success in image synthesis. Artificial GAN-based synthesized images have been widely spread over the Internet with the advancement in generating naturalistic and photo-realistic images. This might have the ability to improve content and media; however, it also constitutes a threat with regard to legitimacy, authenticity, and security. Moreover, implementing an automated system that is able to detect and recognize GAN-generated images is significant for image synthesis models as an evaluation tool, regardless of the input modality. To this end, we propose a framework for reliably detecting AI-generated images from real ones through Convolutional Neural Networks (CNNs). First, GAN-generated images were collected based on different tasks and different architectures to help with the generalization. Then, transfer learning was applied. Finally, several Class Activation Maps (CAM) were integrated to determine the discriminative regions that guided the classification model in its decision. Our approach achieved 100% on our dataset, i.e., Real or Synthetic Images (RSI), and a superior performance on other datasets and configurations in terms of its accuracy. Hence, it can be used as an evaluation tool in image generation. Our best detector was a pre-trained EfficientNetB4 fine-tuned on our dataset with a batch size of 64 and an initial learning rate of 0.001 for 20 epochs. Adam was used as an optimizer, and learning rate reduction along with data augmentation were incorporated.

## 1. Introduction

Image synthesis is the process of generating artificial images from different input modalities, i.e., text, sketch, audio, or image [1]. It is used in many applications, such as art generation [2], photo editing [3,4], photo inpainting [5,6], and computer-aided design [7]. Thus, image synthesis has received intense research, especially after Generative Adversarial Networks (GAN) [8] were developed. GAN has two networks, namely, a generator and a discriminator. The two components compete with each other. While the generator attempts to generate realistic images to fool the discriminator, the discriminator attempts to differentiate between artificial and real images. In the beginning, the generator produces obviously fake data, and the discriminator easily and quickly classifies the generated data as a fake and penalizes the generator for generating implausible data. As the training progresses over the time, the generator learns to produce plausible data that can fool the discriminator, and the discriminator becomes unable to distinguish between synthetic and real data, leading to classifying synthetic data as real. This contest yields in generating high-quality photo-realistic images. Therefore, many researchers have incorporated GANs into image synthesis, which has led to a significant enhancement in generated images. This tremendous progress over the past decade in generating images has led to the provision of synthetic media with a great level of photorealism and quality. This, of course, opens up a great opportunity for artists, designers, movie makers, game designers, and creative users to create new content of interest and enhance their media. However, it may constitute a serious threat regarding legitimacy, authenticity, and security, especially in the hands of malicious users. Malicious users may exploit this advantage to create and disseminate fake media that is well-suited to their attacks. Thus, the media forensic community faces new difficulties as a result of not being able to detect AI-generated images in the real world. Images are considered to be important evidence in forensic investigations that can help in determining the authenticity and origin of sources. Thus, image integrity is critical in forensic investigations. One example of misusing synthetic images in a way that may affect forensic investigations is when generated images are planted at a crime scene or sent to investigation offices as a false lead. Furthermore, being able to create images that may be used for criminal or malicious goals raises a concern. For instance, criminal and malicious users could create fake websites on the internet or fake profiles on social media to widely spread false information or advertise false products that can be used for fraud purposes.

**Figure 1:**
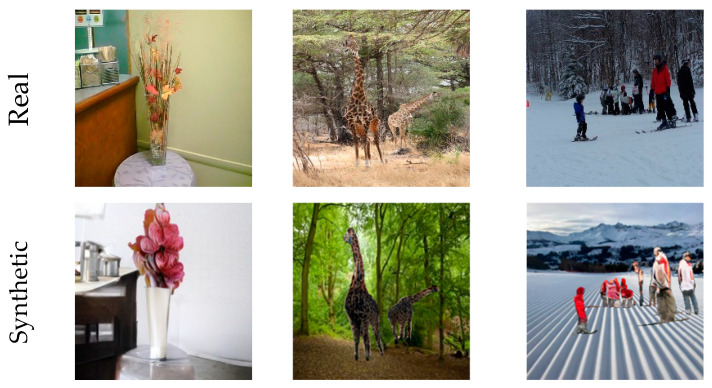
Examples of real images and their corresponding generated ones produced by various GAN-models based on different tasks.

**Figure 2:**
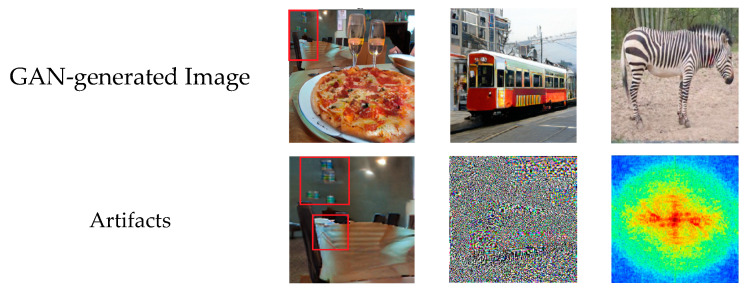
Examples of GAN-synthesized images along with their explicit and implicit artifacts. From left to right: pixel anomalies, artificial fingerprints, and spectral artifacts.

As shown in Figure 1, it is hard to distinguish synthetic images from genuine ones with the rapid improvement in the image synthesis field. Hence, an automated tool for detecting and localizing GAN-generated images is necessary, since most synthetic images contain traces invisible to the human eye. In fact, in spite of the high-quality images resulting from GAN models, synthesized images bear explicit or implicit artifacts resulting from the generation process. Figure 2 shows both explicit artifacts in terms of pixel anomalies and implicit artifacts, which are considered as artificial signatures/fingerprints based on the generative model architecture. Different GAN architectures may have different fingerprints seen in the spatial or frequency domains. Thus, these artifacts could be exploited to distinguish between synthetic and real images. Since each GAN architecture produces a specific pattern that is inserted automatically into each generated image as a distinct fingerprint [9,10], it is hard to generalize detectors on GAN-generated images from other GAN architectures. Other studies have worked on the Fourier domain to detect GAN-generated images from real ones [11,12,13]. These studies have shown that a distinct signature is observed in the frequency domain as a peak, which does not appear in the Fourier spectrum of the real image. This artificial fingerprint depends on the GAN architecture and is caused by the up-sampling operations. Thus, it fails to generalize.

Accordingly, in this paper, we conducted an extensive experimental study that led to creating an automated model that is able to reliably detect GAN-generated images from real ones. Our approach was based on fine-tuning a pre-trained convolutional neural networks (CNN) model on a newly collected dataset. Our dataset was based on different modalities of image synthesis and different GAN architectures to help with the generalization ability across various tasks and architectures. We further visualized where the model looked at the image during the classification process by incorporating various CAMs.

The remainder of this paper is organized as follows. We briefly introduce the related works in Section 2. The proposed data collection and method are presented in Section 3. We conduct the experiments in Section 4 and discuss the results and limitations of our approach in Section 5. Finally, the paper is concluded, and the future work is introduced in Section 6.

## 2. Related Work

Image generation is the task of synthesizing a high-quality realistic image from an image [14,15,16,17], sketch [18], or text [19,20,21,22]. It is a challenging and interesting problem in the computer vision area. Research on image synthesis has been actively conducted over the past decade, especially after the advent of GANs [8]. The GAN architecture and its variants have improved these generated images. Sometimes, these artificial images are hardly distinguishable from real images. This could help in improving the media and content in the digital world; nonetheless, it likewise comprises dangers with respect to authenticity, genuineness, and security.

### 2.1. Image-to-Image Synthesis

This is the process of translating images from a source domain to another domain. This mapping works by modifying some characteristics in the input image from the source domain to have the same characteristics in the target domain while maintaining its content. For instance, segmentation mask maps are transformed into color images [14,15,16,17].

#### 2.1.1. Image-to-Image Synthesis through Conditional GAN (cGAN)

Conditional GAN (cGAN) [23] is a conditional version of GAN, where an additional input, e.g., class labels, is fed into the generator along with the noise input to generate synthetic data. This condition extension, e.g., class labels, is also fed into the discriminator along with the real and synthetic data so it can differentiate between them. OASIS [14] uses a simplified version of the GAN architecture. The discriminator is re-designed as a semantic segmentation network, where label maps are directly used as ground truth. The discriminator’s architecture is based on U-Net [24], which is an encoder–decoder model connected by skip connections. This helps in generating images that are well-aligned with their corresponding semantic label maps. To promote the discriminator to concentrate on the semantic/content and differences in the structural information of the generated and real images, LabelMix regularization is leveraged, leading to re-designing the discriminator. Hence, the generator is re-designed to allow for the synthesis of multi-modal data through 3D noise sampling. This enables partially or completely changing the images, which leads to enhancing the diversity of the generated images. CC-FPSE [15] uses a conditional GAN for semantic image synthesis. It works by exploiting the semantic layout during the generation process. The generator predicts the convolutional kernels restricted on the semantic label maps to produce feature maps. The resulting feature maps from the noise are used to generate the image. To improve the alignment of the synthesized images with their label maps and generate fine details, the discriminator is designed based on a feature pyramid semantic-embedding discriminator. This predicts not only real/fake scores, but also the semantic alignment scores to the semantic label maps. SPADE [16] is proposed upon a pix2pix model [25]. It uses a spatially adaptive normalization layer with learned parameters in the generator to maintain the semantic information, leading to the generation of photo-realistic images. The reason for this is that SPADE normalizes only the activations from the prior layer. Thus, it preserves the input information, as opposed to InstanceNorm [26], which tends to wash away the semantic information during the generation process.

#### 2.1.2. Image-to-Image Synthesis through Transformers

Taming-transformers [17] incorporates a transformer with the inductive bias of a CNN. In the generator, the CNN, which consists of encoder–decoder architecture, learns a discrete spatial codebook. To learn this context-rich codebook and generate local realism, the model uses VQ-GAN, which is a type of VQVAE, along with perceptual loss. Then, the transformer learns the global composition within the image to generate high-resolution images.

### 2.2. Sketch-to-Image Synthesis

This is a task that converts an input sketch into a photo-realistic image. Thus, it maps a simple and rough sketch that only contains basic structural information to a color image that consists of rich features. S2I-DetectoRS [18], S2I-HTC [18], S2I-QueryInst [18], and S2I-MaskRCNN [18] share the same framework and consist of four main stages. In the first stage, a pre-trained instance segmentation model on the MS-COCO dataset [27] is leveraged. Specifically, DetectoRS [28], HTC [29], QueryInst [30], or Mask R-CNN [31] are fine-tuned on different types of edge maps [32,33,34,35] to segment 92 classes of the MS-COCO dataset [27]. Then, the semantic segmentation stage takes place. Next, the resultant semantic mask map is fed into an image-to-image translation model, namely SPADE [16], to generate a photo-realistic image. Finally, a post-processing stage is incorporated to enhance the background further and refine human faces.

### 2.3. Text-to-Image Synthesis

This is the process that maps a natural language description into a realistic image that conveys the same semantic information as the input text [18,19,20,21]. Hence, it substitutes a long sentence into one still image.

#### 2.3.1. Text-to-Image Synthesis though Attention Module

In AttnGAN [19], an attention module is integrated to generate fine details. The words in the input text are encoded into word vectors along with a sentence vector that results from encoding the whole sentence. The generation process occurs over two stages. In the first stage, based on the sentence vector, a low-quality image is generated. Then, multi-stage refinement takes place to successively refine the image by focusing on specific regions of the low-quality image based on the related word vectors. In ControlGAN [22], an attention module is used. In particular, a word-level spatial and channel-wise attention module is integrated into the generator to concentrate on subregions each time, depending on the more related words. Additionally, to obtain fine-grained feedback, a word-level discriminator is introduced to correlate subregions of the image with words. Therefore, this enables the generator to change the visual attributes of specific subregions without impacting the other regions.

#### 2.3.2. Text-to-Image Synthesis though Contrastive Learning

Since the text is created by humans during annotation, different captions might be assigned to one image based on the annotators’ point of view. This discrepancy could lead to generated images which are different from their corresponding ground truth. To tackle this problem, DM-GAN+CL [20] is proposed. It is a contrastive learning method for learning consistent representations of the input text. Hence, this improves the semantic consistency.

#### 2.3.3. Text-to-Image Synthesis though Deep Fusion Block (DFBlock)

DF-GAN [21] is a one-stage, simple, yet effective model that is able to directly generate high-quality images from input text. The generator incorporates a Deep text–image Fusion Block (DFBlock) to enhance the semantic consistency between the synthesized images and their corresponding texts. The discriminator consists of Matching-Aware Gradient Penalty (MA-GP) to encourage the generator to produce more photo-realistic images.

## 3. Data Collection and Methodology

**Figure 3:**
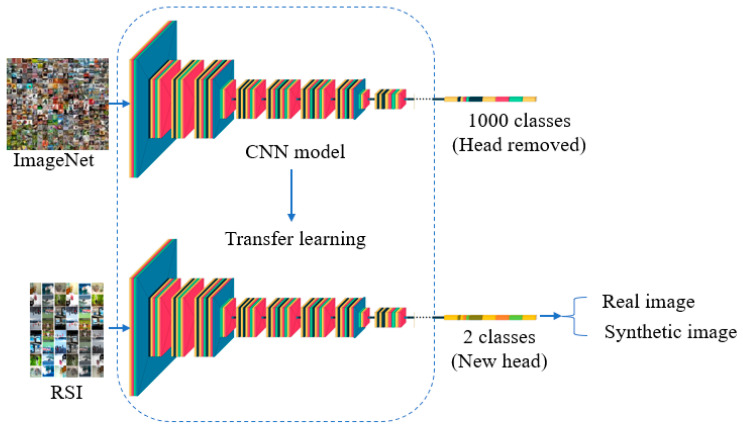
The pipeline of our proposed framework for GAN-generated image recognition.

In this section, we first introduce the Real or Synthetic Images (RSI) dataset that was used for the training and testing. Then, our proposed framework is discussed in detail. An overview of our proposed method is illustrated in Figure 3.

### 3.1. Data Collection: Real or Synthetic Images (RSI)

**Figure 4:**
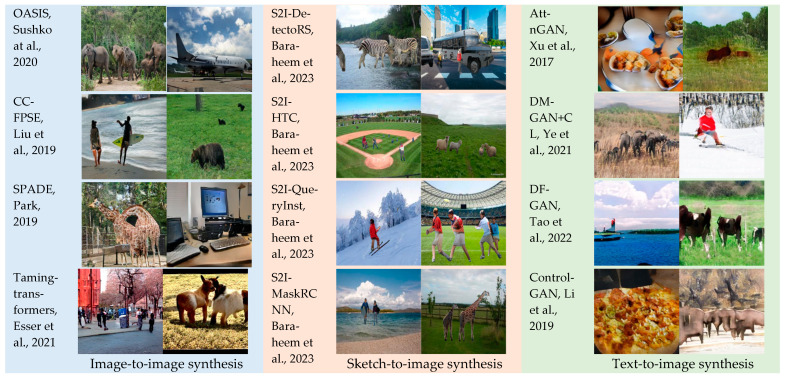
A sample of synthesized images generated by different tasks and different GAN models [14,15,16,17,18,19,20,21,22].

To enable our method to generalize better, a dataset was collected based on 12 different image synthesis models from several tasks, i.e., image-to-image, sketch-to-image, and text-to-image. Having a dataset of various tasks and diverse GAN architectures helped not only with the generalization capability, but also in boosting the accuracy. Our dataset was compiled based on the COCO-Stuff dataset [36] through (1) image-to-image synthesis models: OASIS [14], CC-FPSE [15], SPADE [16], and Taming-transformers [17], (2) sketch-to-image synthesis models: S2I-DetectoRS [18], S2I-HTC [18], S2I-QueryInst [18], and S2I-MaskRCNN [18], and (3) text-to-image synthesis models: AttnGAN [19], DM-GAN+CL [20], DF-GAN [21], and ControlGAN [22]. The COCO-Stuff dataset [36] is an extension of the MS COCO dataset [27]. The COCO dataset [27] contains only instance-level annotations for classes of things. In total, 164k images of the MS COCO dataset [27] were augmented with pixel-level annotations corresponding to stuff. Then, the dataset was split into 118k, 5k, and 40k images for the training, validation, and testing sets, respectively. Indeed, the COCO-Stuff dataset [36] is composed of 172 classes: 80 thing classes, 91 stuff classes, and 1 ‘unlabeled’ class. Thus, it can be used in many tasks, such as classification, detection, segmentation, scene understanding, captioning, and image generation, just to name a few. Examples of synthesized images generated by different tasks conditioned on different input types are shown in Figure 4.

Our dataset consisted of 24k, 12k, and 12k images for the training, validation, and testing, respectively. For each set, the number of images was split evenly between real and synthetic images. More details about our data collection are provided in Table 1.

The process of collecting our dataset was as follows. We first collected real images based on the COCO-Stuff dataset [36]. Then, for each real image, we generated different inputs in terms of text, sketch, and semantic segmentation mask maps depending on [36]. Specifically, for the tasks of text-to-image synthesis and image-to-image synthesis, we collected the captions and semantic mask maps from [36]. However, for the task sketch-to-image synthesis, we generated the sketches based on a dodging and burning algorithm [32], which produced pencil sketches and maintain structural details. Next, for each of the 12 image synthesis models and based on the input modality, we ran the pre-trained model on the generated inputs to obtain synthetic artificial images. The resolution of the real and synthetic images was rescaled to 256 × 256, regardless the image synthesis models’ resolution.

### 3.2. Methodology

With the recent improvements in image synthesis, it is significant to develop an automated tool to dependably and timely recognize GAN-generated images. Thus, after the dataset was collected, we trained a classifier by fine-tuning the classifier that was pre-trained on the ImageNet dataset [37] on our newly collected RSI dataset. The reason for this was that training a classifier from scratch is not only time consuming and costly, but also less efficient in terms of classification performance. Therefore, transfer learning is a better approach. Since there were only two classes in our dataset, namely, real or synthetic, the training process was conducted in binary classification setting. A variety of classifiers were leveraged through an experimental study to reach the best model that was able to reliably and precisely detect and localize GAN-generated images.

The details of the training procedure were as follows. We fine-tuned our dataset on VGG19 [38], ResNet [39] with different number of layers: (50, 101, and 152), InceptionV3 [40], Xception [41], DenseNet121 [42], InceptionResNetV2 [43], MixConv [44], MaxViT [45], and EfficientNetB4 [46]. The head of the classifier pre-trained on ImageNet [37] was removed, and a new head was placed on top of the classifier. The new head for all our classifiers consisted of a global average pooling, a dense layer with a ReLU [47] activation function, a batch normalization layer [48] to reduce the internal co-variant shift and stabilize the training process, a dropout layer [49] to overcome the overfitting problem, and finally, a dense layer with a sigmoid activation function. For all the classifiers, a batch size of 64 was used, and the initial learning rate was 0.001. The training lasted for 20 epochs; however, checkpoints were saved whenever there was an improvement in the validation loss, so that the best model could be loaded later during testing. Moreover, for all the classifiers, the learning rate was reduced automatically when the validation loss stopped improving. Furthermore, data augmentation, in particular, horizontal flip, was applied on the training set during the training process. Adam [50] was incorporated as an optimizer for all the classifiers except ResNet101, where RMSprop [51] was used.

## 4. Results and Analysis

In this section, we first introduce the evaluation metrics used to evaluate performance. Then, the experimental results are reported based on the metrics adopted on our RSI dataset to evaluate the efficiency of our proposed methods in detecting and localizing GAN-generated images from real ones. Next, an ablation study was conducted to show the effectiveness of our proposed approach in recognizing GAN-synthesized images on different modalities of image synthesis (text, sketch, or image) and different GAN-based image synthesis models.

### 4.1. Evaluation Metrics

To evaluate the performance of our proposed methods in detecting GAN-generated images from real ones, eight common evaluation metrics were adopted. Specifically, Precision, Recall, F1 score, Accuracy, Average Precision (AP), Area Under Curve of Receiver Operating Characteristic (ROC-AUC), False Positive Rate (FPR), and False Negative Rate (FNR) were measured and recorded.

### 4.2. Experimental Results on RSI

**Figure 5:**
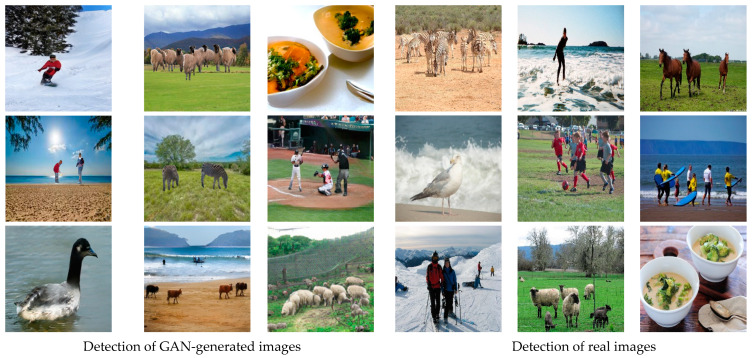
Examples of detecting real and GAN-generated images with 100% accuracy using our best model on the testing set.

A comprehensive performance evaluation was conducted on our RSI dataset to validate the performance of our approach in detecting GAN-generated images. In particular, the testing set of RSI, which consisted of 12,000 images split equally between GAN-generated and real images, based on the COCO-Stuff dataset [36], was used during the evaluation. The eight evaluation metrics were computed on the testing set of our dataset and are reported in Table 2. As can be seen, EfficientNetB4 achieved the best performance in recognizing and localizing GAN-generated images, with a 100% accuracy, followed by InceptionV3 with 98% in terms of accuracy on this particular dataset, i.e., the RSI dataset. Instances of GAN-generated and real images from the RSI dataset that were classified properly via our best model (EfficientNetB4), with a 100% accuracy, are illustrated in Figure 5.

**Figure 6:**
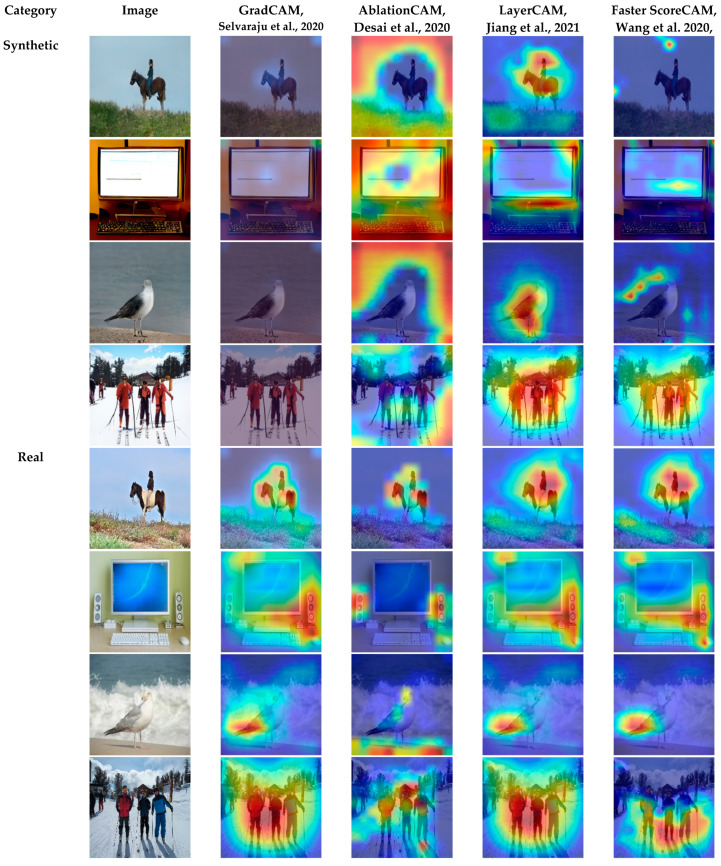
Visualization of variants of CAM on the testing set using our best model EfficientNetB4 [52,53,54,55].

Furthermore, to locate the region where our model was looking at during the classification process, different types of Class Activation Maps (CAM) were integrated. Specifically, GradCAM [52], AblationCAM [53], LayerCAM [54], and Faster ScoreCAM [55] were adopted to inspect the images by identifying which parts of the image contributed more to the classification decision of our model. A visualization of the four different CAM methods is demonstrated in Figure 6.

**Figure 7:**
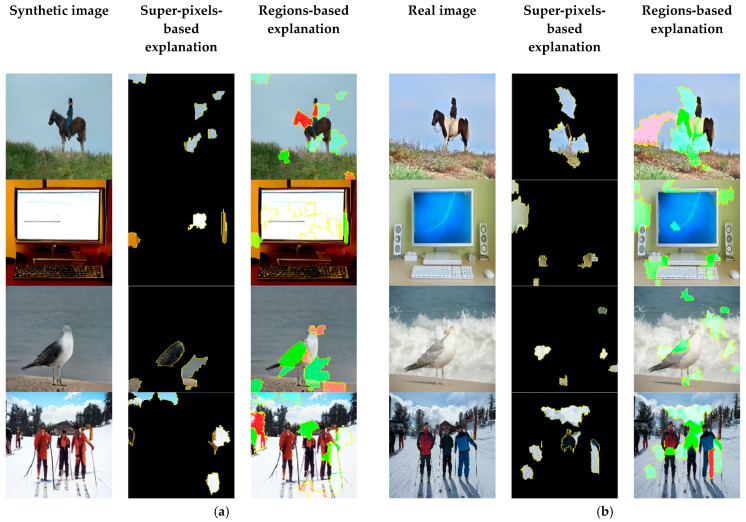
Visualization of LIME interpretability technique on detecting GAN-generated images from real ones based on our method. The first and fourth columns represent the input image whether synthetic or real. The second and fifth columns illustrate LIME explanation based on super-pixels, where only the super-pixels that contribute to the final classification decision are presented. The third and sixth columns demonstrate LIME explanation based on the two regions. While the green-colored regions of super-pixels correspond to regions that increase the probability of the classified label, the red-colored regions of super-pixels correspond to regions that decrease the probability of the classified label. (**a**) GAN-generated images classified as “Synthetic”; (**b**) Real images classified as “Real”.

As shown, the model looked mostly to the background of the GAN-generated images. However, it also looked at some regions where distortion and anomalies appeared in the synthetic images. To faithfully explain the predictions of our method, an explanation technique called LIME [56] was leveraged, as shown in Figure 7.

### 4.3. Effectiveness of Our Model

To show the effectiveness of our model in recognizing GAN-generated images for other models that were not trained on them, we further conducted one more experiment. In this experiment, we trained three models separately based on the input modality. Specifically, we trained a model on sketch-to-image and text-to-image models, named (S2I_T2I). In total, eight models were used for both modalities. Then, the trained model was tested on an image-to-image modality which consisted of four models. The second experiment (I2I_T2I) was that the model was trained on image-to-image and text-to-image models, and then tested on sketch-to-image modality models. The same specification was followed in the third and last experiment, where we trained the model on image-to-image and sketch-to-image models (I2I_S2I), and then tested the trained model on text-to-image models. The results of these experiments are reported in Table 3. As can be seen, while the performance of the S2I_T2I model, when excluding I2I models, achieved 0.99 in terms of accuracy, the I2I_S2I model reached 0.95. In the meantime, the I2I_T2I model achieved less accuracy with 0.83 due to the image improvement step used in sketch-to-image models. This image improvement step attempts to enhance the background by replacing the generated background with a real background consistent and aligned with the context/objects in the image. Even though this step enhances the generated results, our GAN detection model was still able to detect GAN-generated images with a high accuracy of 0.83.

Consequently, our current model proved its capability in detecting GAN-generated images from real ones with a high accuracy, even though the model and its modality were not trained on. Thus, our GAN detection model is able to recognize GAN-generated images if a new GAN model is proposed in the future.

### 4.4. Ablation Study

To demonstrate the effectiveness of our method in detecting GAN-based synthetic images, we evaluated our second-best model, InceptionV3, on the testing set of different input modalities separately. We further assessed the performance of our second-best model on the testing set of image synthesis models individually. The reason that the second-best model, InceptionV3, was used during the ablation study was due to the fact that our best model EfficientNetB4 achieved 100% accuracy. Thus, there were no differences in the performance when part of the dataset was evaluated.

**Figure 8:**
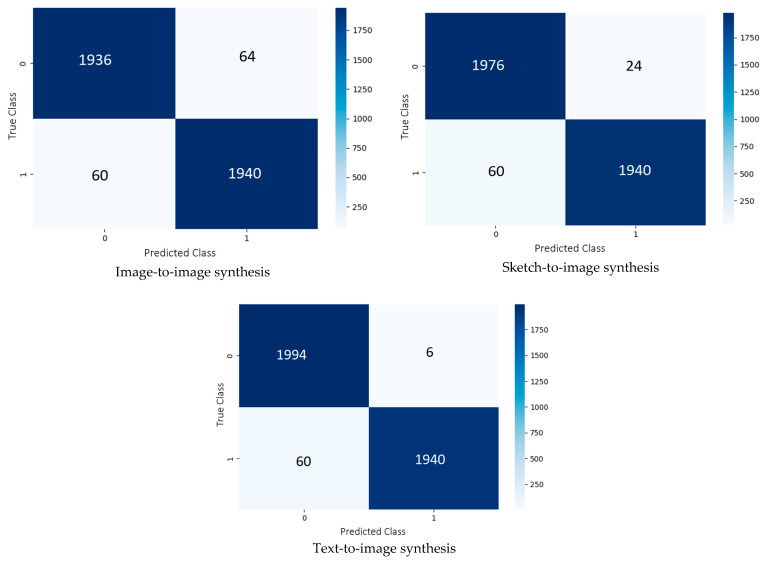
Confusion matrices produced by our second-best model in terms of accuracy (InceptionV3) on the testing set of different input modalities.

While Table 4 reports the performance based on the eight common evaluation metrics, Figure 8 presents the confusion matrices of our second-best model on the testing set generated from the image-to-image, sketch-to-image, and text-to-image synthesis models. The highest accuracy was accomplished with the GAN-based synthetic images generated from the texts, at 98.35%. On the contrary, the lowest accuracy was achieved when the inputs to the image synthesis models were semantic segmentation maps, at 96.90%.

Moreover, we studied the performance of our second-best model on the GAN-synthesized images separately produced by various synthesis images models. Table 5 provides a comprehensive performance evaluation of the models in detecting GAN-generated images produced by a single image synthesis model. From Table 5, we can conclude that it might be more challenging to detect GAN-generated images obtained from semantic segmentation mask maps than ones produced by natural language descriptions. Since the layout, shape, size, and semantic information are maintained in image-to-image synthesis, the generated images are more photo-realistic and naturalistic than the ones produced from text-to-image synthesis.

### 4.5. Experimental Results on Other Datasets

To show our model’s efficacy in detecting generated images from real ones, we further ran one more experiment. In this experiment, the first model in each modality from Table 1 was selected. More specifically, OASIS [14], S2I-DetectoRS [18], and AttnGAN [19] were used as image-to-image, sketch-to-image, and text-to-image synthesis models, respectively. Then, different datasets, which our model was not trained on, were utilized to generate synthetic images. Particularly, the ADE20K [57], Sketchy [58], and Caltech-UCSD Birds-200-2011 (CUB-200-2011) [59] datasets were leveraged. Following this, both generated and real images were fed into our best detector, i.e., EfficientNetB4, to detect synthetic images from real ones. Finally, eight common evaluation metrics were leveraged to measure our model’s performance. Mainly, Precision, Recall, F1 score, Accuracy, Average Precision (AP), Area Under Curve of Receiver Operating Characteristic (ROC-AUC), False Positive Rate (FPR), and False Negative Rate (FNR) were adopted. The details are explained as follows.

With regard to the OASIS image-to-image synthesis model [14], the testing set of the ADE20K dataset [57], which consisted of 2000 semantic segmentation images, was fed into the pre-trained OASIS model [14]. This step output synthesized images from the semantic segmentation images. Then, the generated and real images of the ADE20K testing set [57] were input into our best model, and the performance was evaluated and recorded.

As for S2I-DetectoRS [18], a subset of the Sketchy dataset [58], which contained 2470 sketches, was used as an input into the S2I-DetectoRS sketch-to-image synthesis model [18]. This subset of the Sketchy dataset [58] was used because S2I-DetectoRS [18] was trained on the MS COCO dataset [27], and only 35 classes from the Sketchy dataset [58] matched the classes in the MS COCO dataset [27]. These classes were airplane, alarm_clock, apple, banana, bear, bench, bicycle, car_(sedan), cat, chair, couch, cow, cup, dog, door, elephant, eyeglasses, giraffe, hat, horse, hotdog, knife, motorcycle, pickup_truck, pizza, sailboat, scissors, sheep, shoe, spoon, table, teddy_bear, umbrella, window, and zebra. In the generation phase, sketches were converted into colored images. After that, both generated and real images were fed into our best model, and the eight-evaluation metrics were measured.

With respect to the AttnGAN text-to-image synthesis model [19], the same procedure was followed. The only difference was the dataset, where the testing set of Caltech-UCSD Birds-200-2011 (CUB-200-2011) [59] was used. The testing set was composed of 5794 captions/descriptions. After generating the corresponding images for the input text descriptions, both synthetic and real images were fed into our best model, i.e., EfficientNetB4. In the final step, the performance was assessed based on our adopted evaluation metrics.

The experimental results were recorded and are shown in Table 6. As can be seen, our model was able to detect and recognize the GAN-generated images from real ones, even with other datasets that our classifier was not trained on. While the highest accuracy roughly reached 98% with the CUB-200-2011 dataset [59] and the AttnGAN [18] text-to-image synthesis model, our model achieved 89% in terms of accuracy with the ADE20K dataset [57] and the OASIS [14] image-to-image synthesis model. This may be attributed to the dataset complexity and generator capability. Hence, our model could be used as an evaluation tool regardless of the input modality.

## 5. Discussion and Limitations

This paper provides a comprehensive performance evaluation to assess the performance of our method in GAN-generated images detection. We first evaluated several classifiers that were fine-tuned and re-trained on our newly collected dataset, namely Real or Synthetic Images (RSI). The evaluation process was based on the testing set of RSI that contained 12k images. Based on the evaluation metrics used in this study, EfficientNetB4 achieved the highest accuracy, with 100% on this particular dataset.

To show the efficiency of our model in detecting GAN-generated images, we further conducted two different experiments. The first experiment was based on the input modality, where we fine-tuned and re-trained EfficientNetB4 on two input modalities of the RSI training set. Then, we tested the modality-based trained model on the third excluded input modality of the RSI testing set. Our model achieved a superior performance in terms of accuracy with 99%, 83%, and 95% when I2I, S2I, and T2I were excluded during the training process, respectively. The reason behind the accuracy reduction in our model to 83% when the S2I input modality was excluded could be attributed to the background improvement and face refinement steps used in sketch-to-image models [18]. While the background improvement step works by replacing the synthetic background with a real, high-quality background well-suited and aligned with the foregrounds in each image, the face refinement step works by reconstructing and aligning faces. These post-processing steps improve the synthetic images further. However, our model was still capable of recognizing these images as generated images, even when our model was not trained on the S2I modality, at 83% in terms of accuracy.

In the second experiment, different datasets were leveraged in the evaluation phase to show the ability of our model in detecting GAN-generated images, even with other datasets that our model was not trained on. Based on this experiment, it was clear that our model was able to detect generated images from real ones with a high accuracy. Thus, our model can be used in synthetic images detection, which could help in forensic investigations, mitigating the misuse of AI image generation, the alleviation of cyberattacks, and confronting criminal and malicious goals.

To demonstrate the discriminative region(s) of the image that highly influenced the classifier to make a decision, several types of Class Activation Maps (CAM) were adopted. As seen in Figure 6, our model mostly looked at the background of the generated images. This was because generators usually concentrate more on the foreground than the background, leading to generating explicit or implicit artifacts during the generation process of the background. However, in some cases, our model looked at some parts of the foreground(s) in the generated images. This was because of anomalies and distortions generated in the foreground(s) during the generation process.

**Figure 9:**
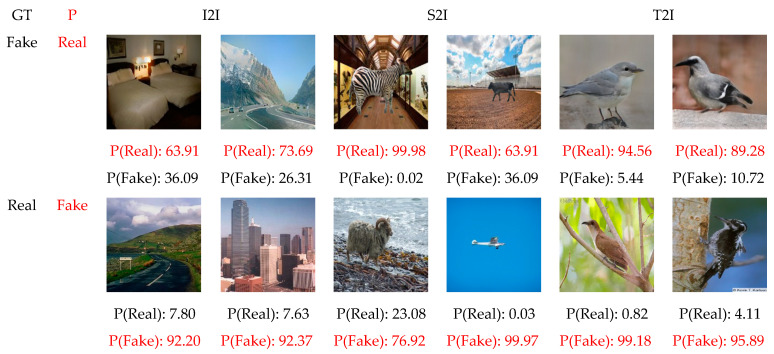
Failure cases classification visualization of our proposed method on different testing datasets, i.e., ADE20K, Sketchy, and CUB-200-2011. While GT indicates the ground truth, P refers to the misclassified prediction obtained by our model, represented in red.

Regardless of the high accuracy produced by our model, it sometimes failed to properly classify images. A visualization of these failure cases is illustrated in Figure 9. As can be seen from this figure, our model might fail in classifying authentic and genuine images as real when the background or foreground(s) is blurry, when the image looks vintage and old, when the image is of low quality, and/or when motion exists in the image. Furthermore, our model could misclassify GAN-generated images as fake when fine-grained details are represented, whether in the foreground(s) or background, and when the textures are sharper, since GAN-generated images tend to have smoother textures.

To overcome these limitations, the aforementioned aspects of misclassifying the images should be more integrated into the training dataset. Thus, our classifier can learn more about these aspects and hence properly classify the images.

## 6. Conclusions and Future Work

With the rapid and continuous evolution of AI-based image synthesis, generated images are coming much closer to being photo-realistic and deceiving human eyes. This may improve media and content; however, it could pose challenges to security and authenticity. Therefore, an automated tool to detect and localize AI-based generated images is necessary. Hence, this paper proposed a machine model for recognizing AI-generated images from real ones. A large dataset, namely, RSI, was compiled based on a variety of GAN-based image synthesis tasks and models, and then a CNN model was well-trained on RSI. Extensive experiments led to a method that is able to recognize GAN-generated images from real ones with an outstanding accuracy, even with other datasets that our model was not trained on. Thus, our detector can be used to detect synthetic images from genuine ones. Hence, it can aid against malicious or criminal ends by helping the multimedia forensics research community to confront threats that may emerge from the advancements in AI image synthesis technologies. Our findings suggest that GAN-generated images contain some common flaws, distortions, and artifacts that can be exploited to detect synthetic images. These traces are not visible to human eyes; however, a well-trained model can easily detect these flaws and classify the images as synthetic/fake images. Therefore, researchers should pay more attention to these traces during the image generation process to generate not only high-quality photo-realistic images, but also to conceal all fingerprints and traces.

In the future, we plan to incorporate different image synthesis tasks with different architectures and datasets, including facial and biological images, e.g., Western blot and microscopic images. Moreover, we aim to integrate the frequency domain of the dataset along with the spatial domain. Finally, to enhance our GAN-generated image detector, we plan to include proper techniques and strategies to overcome possible adversarial attacks added to the synthetic images, where tiny and imperceptible adversarial perturbation added to generated images may fool the detector.

## Figures and Tables

**Table 1:** Specification of our dataset.

Task	Model	Input	Training Set	Validation Set	Testing Set	Total
Image-to-image synthesis	OASIS [14]	Semantic mask map	2000	1000	1000	4000
	CC-FPSE [15]		2000	1000	1000	4000
	SPADE [16]		2000	1000	1000	4000
	Taming-transformers [17]		2000	1000	1000	4000
Sketch-to-image synthesis	S2I-DetectoRS [18]	Sketch	2000	1000	1000	4000
	S2I-HTC [18]		2000	1000	1000	4000
	S2I-QueryInst [18]		2000	1000	1000	4000
	S2I-MaskRCNN [18]		2000	1000	1000	4000
Text-to-image synthesis	AttnGAN [19]	Text	2000	1000	1000	4000
	DM-GAN+CL [20]		2000	1000	1000	4000
	DF-GAN [21]		2000	1000	1000	4000
	ControlGAN [22]		2000	1000	1000	4000
			**24,000**	**12,000**	**12,000**	**48,000**

**Table 2:** Performance of different classifiers on testing set.

	Precision	Recall	F1	Accuracy	AP	ROC-AUC	FPR	FNR
VGG19	0.94	0.94	0.94	0.94	0.9819	0.9803	0.053	0.064
ResNet50	0.93	0.91	0.91	0.91	0.9933	0.9927	0.0035	0.168
ResNet101	0.95	0.95	0.95	0.95	0.9879	0.9877	0.028	0.08
ResNet152	0.92	0.92	0.92	0.92	0.9743	0.9718	0.042	0.118
InceptionV3	0.98	0.98	0.98	0.98	0.9976	0.9974	0.016	0.03
Xception	0.97	0.97	0.97	0.97	0.9995	0.9994	0.0003	0.054
DenseNet121	0.97	0.97	0.97	0.97	0.9969	0.9966	0.012	0.044
InceptionResNetV2	0.96	0.96	0.96	0.96	0.9942	0.9943	0.037	0.036
MixConv	0.94	0.94	0.94	0.94	0.9411	0.9412	0.057	0.056
MaxViT	0.92	0.86	0.89	0.89	0.9375	0.9375	0.087	0.137
**EfficientNetB4**	**1.00**	**1.00**	**1.00**	**1.00**	**1.00**	**1.00**	**0.0**	**0.0**

**Table 3:** Performance of our model trained on two modalities and tested on the third and excluded modality to show the effectiveness of our model in detecting GAN-generated images.

	Precision	Recall	F1	Accuracy	AP	ROC-AUC	FPR	FNR
S2I_T2I	0.99	0.99	0.99	0.99	0.9998	0.9997	0.026	0.001
I2I_T2I	0.87	0.83	0.82	0.83	0.9684	0.9801	0.347	0.0
I2I_S2I	0.96	0.95	0.95	0.95	0.99997	0.99997	0.093	0.0

**Table 4:** Performance of our second-best model in terms of accuracy (InceptionV3) on GAN-generated images individually produced by different input modalities.

	Precision	Recall	F1	Accuracy	AP	ROC-AUC	FPR	FNR
Image-to-image (I2I)	0.97	0.97	0.97	0.9690	0.9961	0.9957	0.032	0.03
Sketch-to-image (S2I)	0.98	0.98	0.98	0.9790	0.9977	0.9974	0.012	0.03
Text-to-image (T2I)	0.98	0.98	0.98	0.9835	0.9991	0.9990	0.003	0.03

**Table 5:** Performance of our second-best model in terms of accuracy (InceptionV3) on GAN-generated images individually produced by different models.

	Input Modality	Precision	Recall	F1	Accuracy	AP	ROC-AUC	FPR	FNR
OASIS [14]	I2I	0.96	0.96	0.96	0.964	0.9958	0.9951	0.042	0.03
CC-FPSE [15]		0.98	0.98	0.98	0.983	0.9983	0.9981	0.004	0.03
SPADE [16]		0.98	0.98	0.98	0.978	0.9979	0.9977	0.014	0.03
Taming-transformers [17]		0.95	0.95	0.95	0.951	0.9928	0.9920	0.068	0.03
S2I-DetectoRS [18]	S2I	0.98	0.98	0.98	0.981	0.9977	0.9974	0.008	0.03
S2I-HTC [18]		0.98	0.98	0.98	0.977	0.9977	0.9973	0.016	0.03
S2I-QueryInst [18]		0.98	0.98	0.98	0.978	0.9977	0.9975	0.014	0.03
S2I-MaskRCNN [18]		0.98	0.98	0.98	0.980	0.9978	0.9974	0.010	0.03
AttnGAN [19]	T2I	0.98	0.98	0.98	0.984	0.9990	0.9989	0.002	0.03
DM-GAN+CL [20]		0.98	0.98	0.98	0.984	0.9996	0.9996	0.002	0.03
DF-GAN [21]		0.98	0.98	0.98	0.982	0.9986	0.9985	0.006	0.03
ControlGAN [22]		0.98	0.98	0.98	0.984	0.9991	0.9990	0.002	0.03

**Table 6:** Performance of our best model on different datasets that our model was not trained on.

	Input Modality	Used Dataset	Precision	Recall	F1	Accuracy	AP	ROC-AUC	FPR	FNR
OASIS [14]	I2I	ADE20K [57]	0.91	0.89	0.89	0.889	0.9839	0.9826	0.008	0.22
S2I-DetectoRS [18]	S2I	Sketchy [58]	0.95	0.94	0.94	0.943	0.9998	0.9998	0.0	0.13
AttnGAN [19]	T2I	CUB-200-2011 [59]	0.98	0.98	0.98	0.978	0.9991	0.9988	0.0	0.04

## Data Availability

The datasets generated during and/or analyzed during the current study are available from the corresponding author on reasonable request.
